# The Deeper Reason

**Published:** 1914-03

**Authors:** 


### The Deeper Reason.

It is painfully evident that the home and society is urgently in need of sex instruction. Besides the terrible toll of the venereal scourge, laid at the door of uncontrolled sex desire, there is still greater toll of bitter disappointment and suffering from matrimonial infelicity, brought on by either abnormal sex relations or over-iudulgence. It is in this class of cases that eugenics is equally interested.

Freud holds that all nervousness and psychoneurotic states are due to an abnormal sex background. He holds that sex tendencies begin in the child, at its mother's breast, that it is manifested in youth except for a few years of repression just preceding adolescence; that during this period of activity should it become perverted or forcibly suppressed, it will not be destroyed, but will remain in the subconscious mind of the individual, there to continue to grow with dynamic force. Later in life when some perversion of the sex function is experienced, this latest force is brought again into consciousness and is so out of tune with the man's moral education that it becomes the most potent factor in causing the psychoneuroses and psychasthenic conditions.

We would not agree with Freud in claiming that all nervousness was due to a perverted sexuality. But to follow the analysis of a case of psychosis one would be impressed with the important part that sex plays in its causation. The writer's recent experience in the psychiatric clinic of Johns Hopkins University would bear out the statements, as fully 90 per cent of the cases seen had a perverted sex background.

Neurasthenia and psychosthenia have increased 150 per cent in thirty years. Impotency in men under thirty is alarming. The growing tendency towards sexual promiscuity and prostitution all point to race deterioration and sounds the call for eugenics.

Dr. Chufford at the recent London Medical Congress in his address on medical progress, says, "That our methods of diagnosis and prognosis should be supplemented by a few notions of collective, family and social progress." "It is the latter," he further stated, "that warrants our legal measures with regard to prophylaxis, and preventive vaccine, and which concurrently with the work of Sir Francis Galton, gave birth to the new science of eugenics, discussed for the first time in London a year ago." Then we see that eugenics is the product of a normal evolution born of sound scientific parentage. Come as a salvation to future generations.

